# BMSC Transplantation Aggravates Inflammation, Oxidative Stress, and Fibrosis and Impairs Skeletal Muscle Regeneration

**DOI:** 10.3389/fphys.2019.00087

**Published:** 2019-02-13

**Authors:** Xiaoguang Liu, Lifang Zheng, Yongzhan Zhou, Yingjie Chen, Peijie Chen, Weihua Xiao

**Affiliations:** School of Kinesiology, Shanghai University of Sport, Shanghai, China

**Keywords:** bone marrow mesenchymal stem cells, skeletal muscle, regeneration, inflammation, oxidative stress, fibrosis

## Abstract

Skeletal muscle contusion is one of the most common muscle injuries in sports medicine and traumatology. Bone marrow mesenchymal stem cell (BMSC) transplantation has been proposed as a promising strategy to promote skeletal muscle regeneration. However, the roles and underlying mechanisms of BMSCs in the regulation of skeletal muscle regeneration are still not completely clear. Here, we investigated the role of BMSC transplantation after muscle contusion. BMSCs were immediately transplanted into gastrocnemius muscles (GMs) following direct contusion. Comprehensive morphological and genetic analyses were performed after BMSC transplantation. BMSC transplantation exacerbated muscle fibrosis and inflammation, as evidenced by increased leukocyte and macrophage infiltration, increased inflammatory cytokines and chemokines, and increased matrix metalloproteinases. BMSC transplantation also increased muscle oxidative stress. Overall, BMSC transplantation aggravated inflammation, oxidative stress and fibrosis and impaired skeletal muscle regeneration. These results, shed new light on the role of BMSCs in regenerative medicine and indicate that caution is needed in the application of BMSCs for muscle injury.

## Introduction

Severe skeletal muscle injuries are commonly observed inclinics of sports medicine and traumatology (Liu et al., 2018). However, there are no effective strategies for treating skeletal muscle injuries. Conservative treatment, such as “rest, ice, compression and elevation” are insufficient for muscle injury repair (Natsu et al., 2004), and muscle fibrosis and dysfunction are commonly observed after severe muscle injury, such as contusion, in patients following a conservative treatment protocol (Xiao et al., 2016b).

Recently, stem cell transplantation has been proposed as a promising treatment for various muscle diseases, such as skeletal muscle injury (Aldahmash et al., 2012; Farjah et al., 2018). Muscle satellite cells are generally used as a source of skeletal muscle stem cells to treat muscle injury or muscle dystrophy (Bashir et al., 2014). However, skeletal satellite cells are relatively rare in skeletal muscle tissue, and they often lose their myogenic potential after *in vitro* expansion (Sassoli et al., 2012). BMSCs have higher proliferative potential and pluripotency and lower rates of donor site morbidity than common satellite cells (Winkler et al., 2009).

Bone marrow mesenchymal stem cells can also effectively differentiate into skeletal muscle cells both *in vivo* and *in vitro* (Galli et al., 2014). Several studies have demonstrated that transplantation of mesenchymal stem cells derived from bone marrow promotes muscle regeneration and accelerates the functional recovery of injured skeletal muscle (Winkler et al., 2008; von Roth et al., 2012b, 2013). However, the mechanism responsible for the beneficial effects on in skeletal muscle regeneration after transplantation of BMSCs remains to be investigated. Moreover, BMSCs have been used to treat muscle atrophy (Geng et al., 2009), toxicant injection-induced muscle injury (Dezawa et al., 2005; de la Garza-Rodea et al., 2011), traumatic muscle injury (Merritt et al., 2010), crush trauma (Winkler et al., 2012), and laceration (Natsu et al., 2004). Here, we investigated the role of BMSCs in regulating skeletal muscle regeneration after contusion.

## Materials and Methods

### Animals

Eighty-eight male C57BL/6J mice weighing 18.1–21.3 g at 7 weeks of age were obtained from Shanghai Jiesijie Laboratory Animal Co., Ltd. After acclimatization to the local environment for 1 week, the mice were divided into the following three groups: normal control mice without muscle injury (group 1), muscle contusion mice treated with vehicle (group 2), and muscle contusion mice treated with BMSCs (group 3). The animals were housed at a constant temperature of 25°C with free access to pellet food and water. The study was approved by the Ethics Review Committee for Animal Experimentation of the Shanghai University of Sport, Shanghai, China (reference number 2016006).

### Isolation and Culture of BMSCs

Tibia and femur bones were harvested from male C57BL/6J male mice. Bone marrow was flushed from the tibia and femur bones with DMEM complete medium. Cells were cultured without disturbance for 24 h, were washed to remove non-adherent cells, and were supplied with fresh DMEM complete medium, with medium renewal every 3 days (Leroux et al., 2010; Su et al., 2014).

### Generation of Mouse Hind Limb Injury

The mice were anesthetized with 400 mg/kg chloral hydrate administered intraperitoneally. The hind limb contusion was operatively induced as previously described with a simple pendulum device. Briefly, the hind limb was positioned by extending the knee and plantarflexing the ankle to 90°. A 16.8 g (diameter, 15.9 mm) stainless steel ball was dropped from a height of 125 cm through a tube (interior diameter of the tube, 16 mm) onto an impactor with a surface of 28.26 mm^2^, resting on the middle of the gastrocnemius muscle (GM) of the mice. The muscle contusion created by this method was a high-energy blunt injury that created a large hematoma, which was followed by muscle regeneration, a healing process that is very similar to that observed in humans (Liu et al., 2016, 2018; Xiao et al., 2016a).

### BMSCs Intramuscular Injection

Bone marrow mesenchymal stem cells were collected, washed twice in PBS, and resuspended in PBS. Either 1 × 10^6^ BMSCs or PBS was injected into the injured muscle. Cell injections were performed with a 27-gauge needle immediately after muscle injury by direct intramuscular injection into the middle point of the gastrocnemius muscle. The GMs were harvested from the mice 3, 6, 12, and 24 days after the treatment for further analyses (Leroux et al., 2010).

### Flow Cytometry

Flow cytometry was performed on a Cytomics^TM^ FC 500 System (Beckman Coulter) using a blue laser (488 nm). The culture medium was removed, and BMSCs were washed twice resuspended in PBS at a concentration of 1x10^5^ cells/mL, and stained with the following monoclonal antibodies: CD29-phycoerythrin (PE), CD44 (PE), at a concentration of 0.2 mg/mL, CD11b (FITC) and CD45 (FITC), at a concentration of 0.5 mg/mL, and isotype controls for FITC and PE (both from Biolegend, San Diego, CA, United States). Cells were incubated in the dark for 30 min at room temperature. The cells were washed with 2 mL of PBS and resuspended in 300 μL of PBS for image acquisition. A minimum of 10,000 events were counted for each analysis.

### Hematoxylin and Eosin (H&E) Staining

Skeletal muscle sections (8 μm) were cut from the mid-belly region of the gastrocnemius muscle and stained with H&E to evaluate the general morphology of the skeletal muscle regeneration.

### Masson’s Trichrome Staining

Masson’s trichrome staining was used to measure the area of fibrotic tissue in the injured skeletal muscle. The collagen fibers were stained blue, the nuclei were stained black, and the background was stained red. After this staining procedure, the ratio of the fibrotic area to the total cross-sectional area was calculated to estimate fibrosis formation using ImageJ 1.44 (NIH, Bethesda, MD, United States).

**Table 1:** Primers used for qRT-PCR.

Target gene	Forward primer sequences	Reverse primer sequences
CD68	5′-CAAAGCTTCTGCTGTGGAAAT-3′	5′-GACTGGTCACGGTTGCAAG-3′
F4/80	5′-AACATGCAACCTGCCACAAC-3′	5′-TTCACAGGATTCGTCCAGGC-3′
TNF-α	5′-CTTCTGTCTACTGAACTTCGGG-3′	5′-CACTTGGTGGTTTGCTACGAC-3′
INF-γ	5′-GCTTTGCAGCTCTTCCTCAT -3′	5′-GTC ACC ATCCTTTTGCCAGT -3′
IL-1β	5′-TGACGTTCCCATTAGACAACTG -3′	5′-CCGTCTTTCATTACACAGGACA-3′
TGF-β	5′-TGCGCTTGCAGAGATTAAAA-3′	5′-CGTCAAAAGACAGCCACTCA-3′
IL-6	5′-GAACAACGATGATGCACTTGC-3′	5′-CTTCATGTACTCCAGGTAGCTATGGT-3′
Col1a1	5′-GAGCGGAGAGTACTGGATCG-3′	5′-GCTTCTTTTCCTTGGGGTTC-3′
Col3a1	5′-GTCCACGAGGTGACAAAGGT-3′	5′-GATGCCCACTTGTTCCATCT-3′
MMP-1	5′-AGTTGACAGGCTCCGAGAAA-3′	5′-CACATCAGGCACTCCACATC-3′
MMP-2	5′-ACCCTGGGAGAAGGACAAGT-3′	5′-ATCACTGCGACCAGTGTCTG-3′
MMP-9	5′-CGTCGTGATCCCCACTTACT-3′	5′-AACACACAGGGTTTGCCTTC-3′
MMP-10	5′-GAGTGTGGATTCTGCCATTGA-3′	5′-TCTCCGTGTTCTCCAACTGC-3′
MMP-14	5′-CCTGGCTCATGCCTACTTCC-3′	5′-GCACAGCCACCAAGAAGATG-3′
CCL2	5′-GCTCAGCCAGATGCAGTTAAC-3′	5′- CTCTCTCTTGAGCTTGGTGAC-3′
CCR2	5′-GAAAAGCCAACTCCTTCATCAG-3′	5′-TCTAAGCACACCACTTCCTCTG-3′
CCL5	5′-CATATGGCTCGGACACCA-3′	5′-ACACACTTGGCGGTTCCT-3′
CXCR4	5′- CAAGGCCCTCAAGACGACAG-3′	5′- CCCCCAAAAGGATGAAGGAG-3′
TWEAK	5′-GCTCCCAAAGCCCCTACTTAT-3′	5′-AGGTCCAGCCCATCTCAGT-3′
Fn14	5′-GGCGCTCTTAGTCTGGTCCT-3′	5′-TGGATCAGTGCCACACCTG-3′
gp91phox	5′-CCAGTGAAGATGTGTTCAGCT-3′	5′-GCACAGCCAGTAGAAGTAGAT-3′
GAPDH	5′-ACTCCACTCACGGCAAATTC-3′	5′-TCTCCATGGTGGTGAAGACA-3′

### Real-Time Polymerase Chain Reaction

The total RNA of the skeletal muscle was isolated using a modified guanidinium isothiocyanate-CsCl method (Liu et al., 2018). RNA was reverse transcribed into cDNA using a commercially available kit (RevertaidTM First Strand cDNA Synthesis Kit, Thermo Scientific). Quantitative PCR was carried out in triplicate via reactions utilizing 10 μL of 2 × Maxima SYBR Green/ROX qPCR Master mix (Vazyme), 1 μL of cDNA, nuclease-free water and 300 nM of each primer (Table 1).

### Statistical Analysis

The data were presented as the mean ± SD and analyzed using SPSS 20.0. The mean values of the genetic data were compared using repeated-measure analysis. *Post hoc* multiple comparisons were performed using the Bonferroni test. The data of the scar tissue area were compared using an independent samples *t*-test. Differences between values were considered statistically significant when P-values were less than 0.05.

**FIGURE 1:**
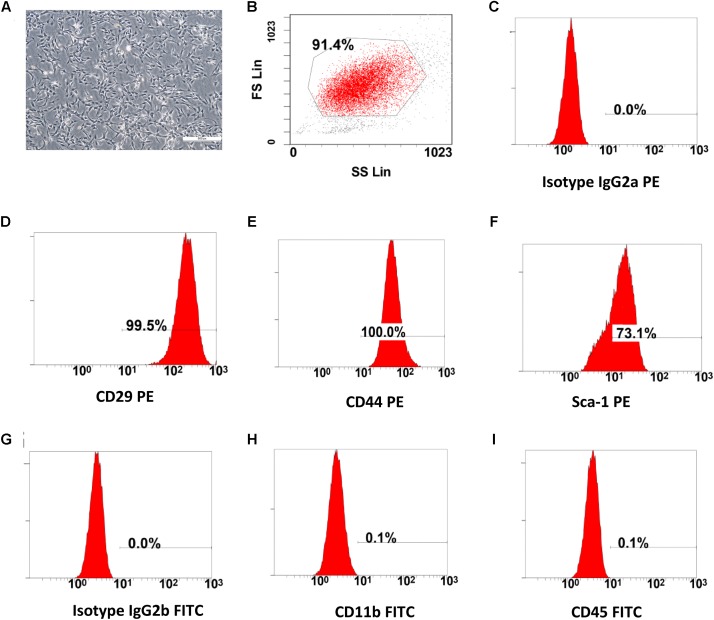
The characterization of BMSCs. **(A)** Cultured bone marrow mesenchymal stem cells, after three passages (scale bar: 100 μm). **(B–I)** Flow cytometry (FC) analysis for cell surface antigens. The morphology (fibroblast-like cell) and cell-surface marker (CD29, CD44, Sca-1 High and CD11b, CD45 Low) of the cultured BMSCs were compliant with the standards of mesenchymal stem cells.

## Results

### The Characterization and Cell Surface Antigens of BMSCs

The cultured BMSCs were fibroblast-like cells and the BMSCs formed homogenous colonies. Most of the BMSCs were had clear cellular boundaries (Figure 1A). FC analysis showed that BMSCs had high expression levels of CD29, CD44, Sca-1 and low expression of CD11b and CD45 (Figure 1B–I). The general morphological characteristics and the expression of relevant cell surface markers were consistent with the criteria used to define mesenchymal stem cells by the International Society for Cellular Therapy (ISCT) (Dominici et al., 2006; Carvalho et al., 2008).

**FIGURE 2:**
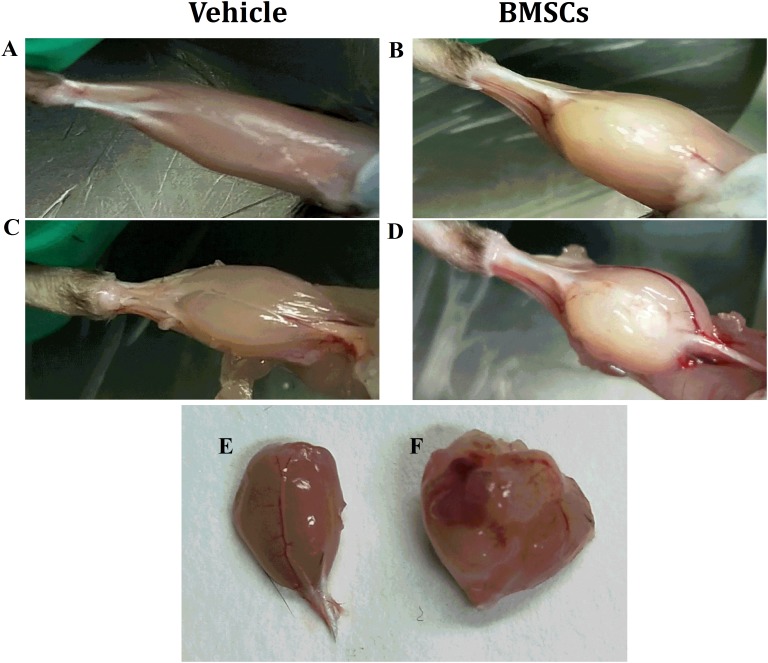
Macroscopic appearances of GMs after BMSCs were treated. **(A)** Vehicle treated GMs at 24 days post-injury; **(B)** BMSCs treated GMs at 24 days post-injury; **(C)** Vehicle treated and fascia stripped GMs at 24 days post-injury; **(D)** BMSCs treated and fascia stripped GMs at 24 days post-injury; **(E)** harvested GMs with vehicle treated at 24 days post-injury; **(F)** Harvested GMs with BMSCs treated at 24 days post-injury; Irregular lumps were found in the BMSCs treated mice other than the vehicle treated mice.

**FIGURE 3:**
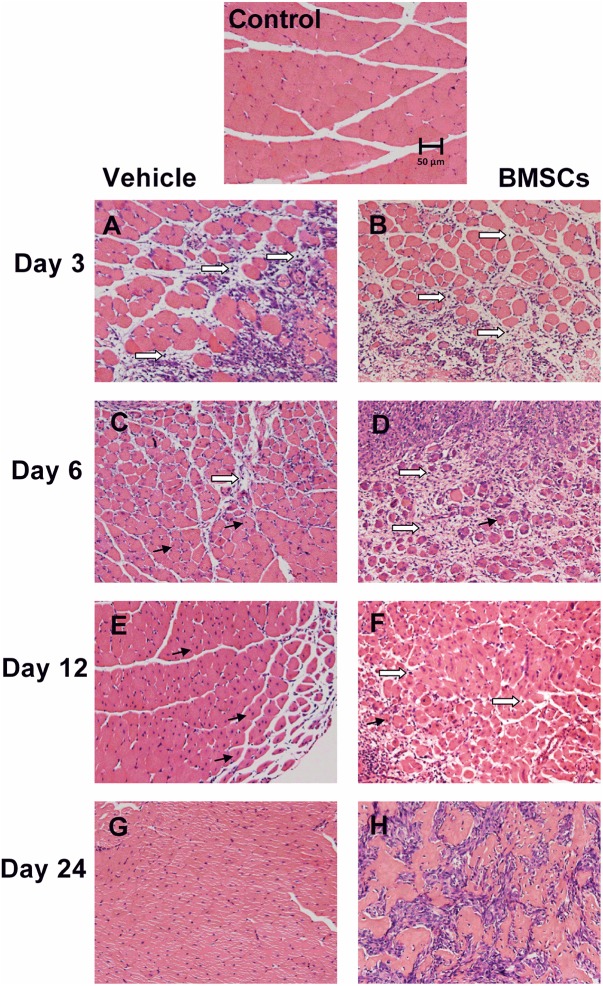
Transplantation of BMSCs impaired skeletal muscle regeneration. Control, uninjured skeletal muscle; Vehicle, vehicle treated group after muscle contusion; BMSCs, BMSCs treated group after muscle contusion. Scale bars, 50 μm; → centronucleated muscle fiber; ⇒ inflammatory cells. **(A–H)** Different time after skeletal muscle injury. There were more inflammatory cells, necrotic myofibers and less central-nucleated regenerated myofibers as compared with the vehicle treated mice, and BMSCs transplation impair muscle regeneration.

### Transplantation of BMSCs Impaired Skeletal Muscle Regeneration

We found that the BMSC treated mice showed irregular lumps in their GMs, while irregular lumps were absent in the vehicle-treated group. In addition, the gastrocnemius muscle mass of the BMSCs treated mice was significantly larger than that of the gastrocnemius muscle collected from the vehicle-treated mice (Figure 2A–F).

Hematoxylin and eosin staining was performed to evaluate whether BMSC transplantation improved skeletal muscle regeneration after injury. H&E staining showed that the GMs from the BMSCs treated mice 3 and 6 days after injury had significantly less central-nucleated regenerating muscle fibers and significantly more infiltrated leukocytes compared with the GMs collected from the corresponding vehicle treated mice. In addition, H&E staining showed that the GMs from BMSC-treated muscle does not exhibit improved morphology of the injured skeletal muscle. Rather, the BMSC treatment may have impaired muscle regeneration after contusion, as there were fewer central-nucleated regenerating myofibers and more inflammatory cells in the BMSC-treated group compared with the vehicle group 3 and 6 days after muscle injury (Figure 3A–D). In addition, at 12 and 24 days post-injury, the damaged area in the vehicle group had been replaced mostly by intact skeletal muscle fibers, whereas numerous necrotic myofibers dominated the injured muscle regions of the BMSC-treated mice (Figure 3E–H).

### Transplantation of BMSCs Aggravated the Fibrosis of Contused Muscles

**FIGURE 4:**
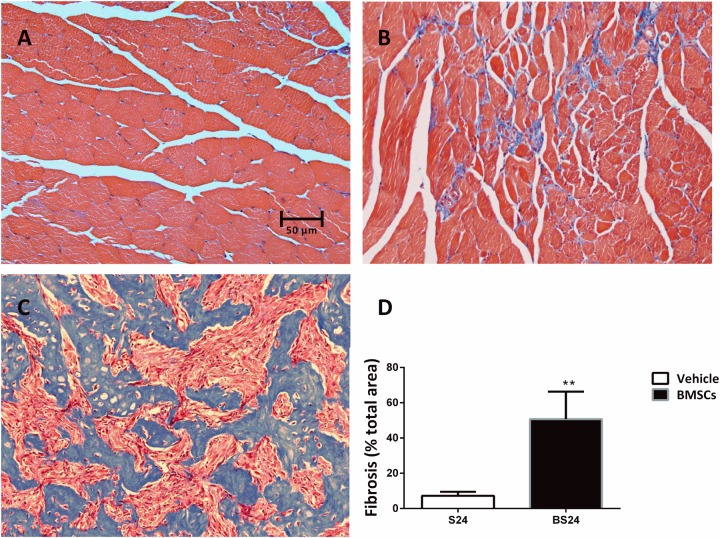
Representative images of fibrosis formation in injured GMs. **(A)** Control muscle; **(B)** Vehicle treated group (24 days post-injury); **(C)** BMSCs treated group (24 days post-injury); **(D)** Quantification of the scar tissue area in injured GMs. Scale bars = 50 μm. Data are means ± S.D., *n* = 6. ^∗∗^ Significant difference from S24, ^∗∗^*P* < 0.01. The BMSCs treated mice showed more fibrosis than the vehicle treated mice at 24 days post-injury.

**FIGURE 5:**
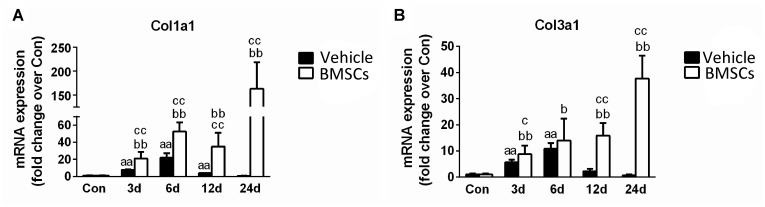
The effects of BMSCs treatment on the expression of collagen. **(A)** The expression of mRNA of Col1a1. **(B)** The expression of mRNA of Col3a1. Vehicle, muscle contusion and vehicle treated group; BMSCs, muscle contusion and BMSCs treated group. Data are means ± S.D., *n* = 8; ^a^ Significant difference from Control, *P* < 0.05; ^aa^*P* < 0.01. ^b^Significant difference from control, *P* < 0.05; ^bb^*P* < 0.01. ^c^Significant difference from the same time points of group vehicle, ^c^*P* < 0.05; ^cc^*P* < 0.01. The transplantation of BMSCs significantly increased the expression of collagen I and III mRNA as compared with the vehicle treated mice after muscle contusion.

The BMSC-treated skeletal muscle showed significantly more fibrosis than the vehicle group 24 days after the contusion injury (7.23 ± 2.26 vs. 50.73 ± 15.5, *p* < 0.01) (Figure 4). Moreover, we used RT-PCR to test the expression of collagen I and III. As expected, the examination revealed that BMSC transplantation significantly increased the expression of collagen I mRNA levels at 3, 6, 12, and 24 days post-injury (*p* < 0.01) compared with the collagen I mRNA levels of the vehicle group (Figure 5A). The expression of collagen III mRNA in the BMSCs-treated group also increased significantly 3, 12, and 24 days after the contusion injury compared with the collagen III mRNA levels of the vehicle group (Figure 5B).

### Transplantation of BMSCs Increased the Expression of Specific Markers of Macrophages in Contused Muscles

**FIGURE 6:**
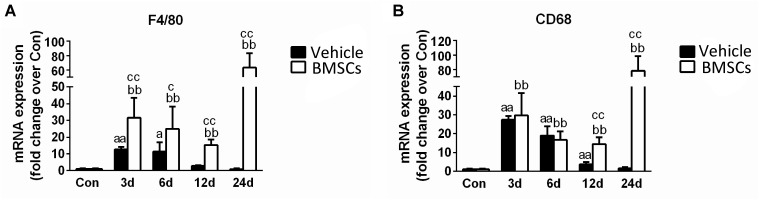
The effects of BMSCs treatment on the expression of macrophages. Vehicle, muscle contusion and vehicle treated group; BMSCs, muscle contusion and BMSCs treated group. **(A)** The expression of F4/80 mRNA after skeletal muscle injury. **(B)** The expression of CD68 mRNA after skeletal muscle injury. Data are means ± S.D., *n* = 8; ^a^Significant difference from control, *P* < 0.05; ^aa^*P* < 0.01. ^b^ Significant difference from control, *P* < 0.05; ^bb^*P* < 0.01. ^c^Significant difference from the same time points of group vehicle, ^c^*P* < 0.05; ^cc^*P* < 0.01. F4/80, the specific marker of macrophage membrane; CD68, the specific marker of macrophages. Specific marker of macrophage (F4/80 and CD68) were significantly increase in the BMSCs treated mice as compared with the vehicle treatd mice in the later stage (12–24 days) of muscle regeneration.

F4/80 a mouse macrophage-specific membrane marker (Starkey et al., 1987). The RT-PCR data showed that F4/80 mRNA increased significantly at the early stage of regeneration, especially in the first 6 days (Figure 6A). In addition, the expression of F4/80 mRNA increased in the BMSC-treated group at 3, 6, 12, and 24 days post-injury (*P* < 0.05). BMSC transplantation increased the expression of CD68, a macrophage-specific endosomal protein (da Silva and Gordon, 1999), at 12 days (*p* < 0.01) and 24 days (*p* < 0.01) after muscle contusion compared with the CD68 expression of the vehicle group (Figure 6B).

### Transplantation of BMSCs Increased the Expression of Inflammatory Cytokines in Contused Muscles

**FIGURE 7:**
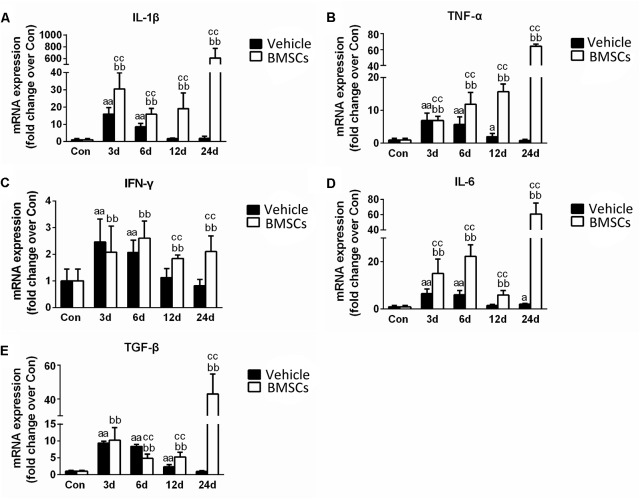
The effects of BMSCs treatment on the expression of inflammatory cytokines. Vehicle, muscle contusion and Vehicle treated group; BMSCs, muscle contusion and BMSCs-treated group. **(A–E)** The expression of inflammatory cytokines after skeletal muscle injury. Data are means ± S.D., *n* = 8; ^a^Significant difference from control, *P* < 0.05; ^aa^*P* < 0.01. ^b^ Significant difference from control, *P* < 0.05; ^bb^*P* < 0.01. ^c^Significant difference from the same time points of group vehicle, ^c^*P* < 0.05; ^cc^*P* < 0.01. BMSCs transplantation enhanced the inflammatory response in skeletal muscle regeneration.

Real-time polymerase chain reaction demonstrated that the expression levels pro-inflammatory cytokines (such as TNF-α, IL-1β, IFN-γ, IL-6, and TGF-β) significantly increased in the early stage of regeneration. With the exception of IL-6, the mRNA levels of these pro-inflammatory cytokines returned to normal 24 days post-injury. BMSC treatment significantly exacerbated the increases in these pro-inflammatory cytokines (such as TNF-α, IL-1β, IFN-γ, IL-6, and TGF-β), indicating that BMSC transplantation enhanced the inflammatory response in skeletal muscle regeneration (Figure 7).

### Transplantation of BMSCs Increased the Expression of Chemokines in Contused Muscles

**FIGURE 8:**
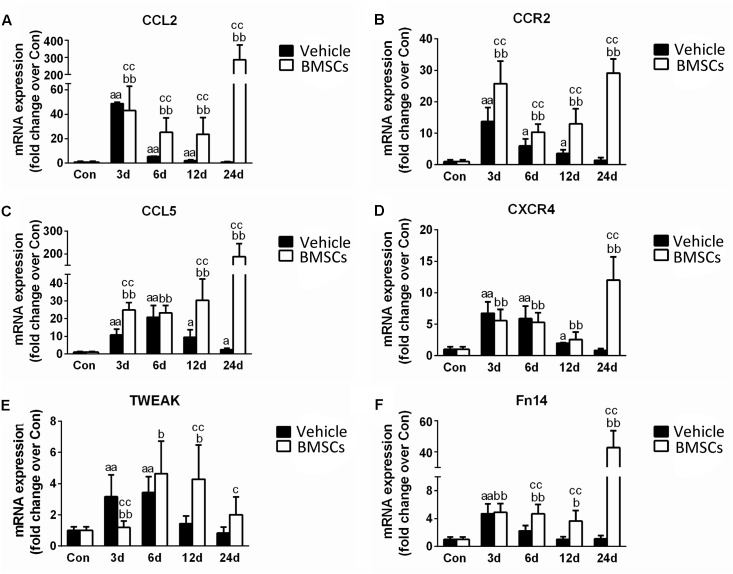
The effects of BMSCs treatment on the expression of chemokines. Vehicle, muscle contusion and vehicle treated group; BMSCs, muscle contusion and BMSCs treated group. **(A–F)** The expression of chemokines after skeletal muscle injury. Data are means ± S.D., *n* = 8; ^a^Significant difference from control, *P* < 0.05; ^aa^*P* < 0.01. ^b^Significant difference from control, *P* < 0.05; ^bb^*P* < 0.01. ^c^Significant difference from the same time points of group vehicle, ^c^*P* < 0.05; ^cc^*P* < 0.01. BMSCs transplantation significantly enhanced the expression of CCL2, CCR2, CCL5, CXCR4, TWEAK, and Fn14 in injured gastrocnemius muscle.

To understand the mechanism underlying the increased leukocyte infiltration after BMSC transplantation, we further analyzed skeletal muscle chemokines. Compared with the control group, the injured skeletal muscle showed increased content of CCL2, CCR2, CCL5, CXCR4, TWEAK, and Fn14 at the early stage of regeneration. With the exception of CCL5, the above chemokines returned to normal at 24 days post-injury. Compared with vehicle treated groups, the BMSC-treated group exhibited significantly enhanced expressions of CCL2 (297.85-fold, *p* = 0.001), CCR2 (20.51-fold, *p* = 0.001), CCL5 (76.16-fold, *p* = 0.001), CXCR4 (14.14-fold, *p* = 0.001), TWEAK (2.39-fold, *p* = 0.001) and Fn14 (38.85-fold, *p* = 0.001) in injured gastrocnemius muscle at 24 days post-injury (Figure 8A–F).

### Transplantation of BMSCs Increased the Expression of Matrix Metalloproteinase in Injured Skeletal Muscles

**FIGURE 9:**
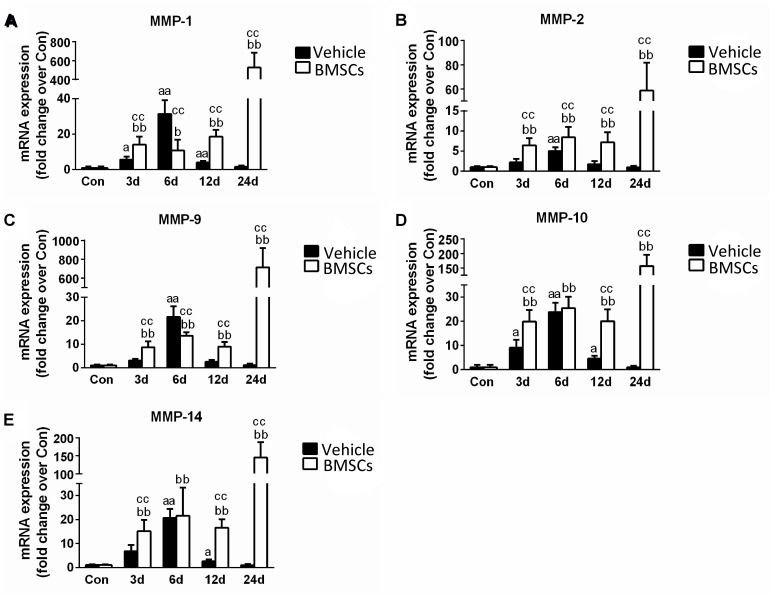
The effects of BMSCs treatment on the expression of matrix metalloproteinase. Vehicle, muscle contusion and vehicle treated group; BMSCs, muscle contusion and BMSCs treated group. **(A–E)** The expression of MMPs after skeletal muscle injury. Data are means ± S.D., *n* = 8; ^a^Significant difference from control, *P* < 0.05; ^aa^*P* < 0.01. ^b^Significant difference from control, *P* < 0.05; ^bb^*P* < 0.01. ^c^Significant difference from the same time points of group vehicle, ^c^*P* < 0.05; ^cc^*P* < 0.01. Transplantation of BMSCs resulted in significant more increases of MMPs in injured gastrocnemius muscle.

The skeletal muscle injury caused significant increases in muscle MMP-1 (31.43-fold), MMP-2 (5.03-fold), MMP-9 (21.62-fold), MMP-10 (23.87-fold), and MMP-14 (20.66-fold) 6 days after injury, which returned to normal at 24 days post-injury. The transplantation of BMSCs resulted in significantly greater increases in MMP-1, MMP-2, MMP-9, MMP-10, and MMP-14 at 12 and 24 days (*p* < 0.01) post-injury (Figure 9A–E).

### Transplantation of BMSCs Increased the Expression of NADPH Oxidases in Injured Skeletal Muscles

Gp91phox is a key subunit of NADPH oxidases and often used as a marker of NADPH oxidases (Xiao et al., 2013). RT-PCR data showed that gp91phox mRNA levels were significantly increased at 3, 6, and 12 days post-injury. Compared with the vehicle treated groups, transplantation of BMSCs resulted in a significant increase in gp91phox at 3 days (1.98-fold, *p* = 0.001), 6 days (2.35-fold, *p* = 0.001), 12 days (4.98-fold, *p* = 0.001) and 24 days (34.17-fold, *p* = 0.001) post-injury (Figure 8).

## Discussion

Skeletal muscle contusion is a common muscle injury in humans, which is particularly common in sport activities and high speed vehicle accidents. It is important to develop effective methods to treat muscle injuries (Liu et al., 2016). Although muscles have a strong regenerative ability, the severity of the injury (such as contusion) might prevent complete regeneration. BMSCs have been intensively studied in the past decade as a promising therapy for many diseases. BMSCs transplantation has been proposed as a treatment for skeletal muscle injury. Although no agreement has been reached on the clinical applications of BMSCs, some reports have generated high expectations for this kind of therapeutic approach (Carvalho et al., 2008). Several studies have found the application of BMSCs to treat muscle injury, although the exact mechanisms leading to this process remain to be elucidated.

The original aim of this study was to investigate the role of BMSC transplantation in muscle regeneration after contusion. We found that BMSC transplantation impaired skeletal muscle regeneration.

Macroscopic appearances and H&E staining revealed that BMSCs treated mice showed irregular lumps, and numerous necrotic myofibers dominated the injured muscle regions after contusion. Furthermore, the Masson’s trichrome staining results showed that the BMSC-treated mice exhibited significantly more fibrosis than the vehicle treated mice 24 days after the contusion injury (Figure 4). As is the case in most tissues, the major ECM muscle protein was collagen, of which the type I and type III isoforms dominated (Lieber and Ward, 2013). Fibrosis was demonstrated by large increases in collagen I and III in the muscle ECM (Huebner et al., 2008). Consistent with the Masson’s trichrome staining results, levels of collagen I and III increased significantly in the BMSC-treated group compared with the vehicle group. Taken together, these findings suggest that transplantation of BMSCs impairs skeletal muscle regeneration and aggravates muscle fibrosis after contusion. Our results suggest that BMSC transplantation is a double-edged sword in injured skeletal muscle and that inappropriate transplantation impairs skeletal muscle regeneration. This viewpoint has been tested in other disease models, such as lung injury (Yao et al., 2018), and various cancers (Norozi et al., 2016; Lee and Hong, 2017) including leukemic (Low et al., 2015) and hepatocarcinoma (Zong et al., 2018).

To explore the mechanism related to BMSC transplantation that impairs skeletal muscle regeneration, we tested the expression of specific markers of macrophages. Macrophages play complex roles in injured skeletal muscle and are involved in muscle fibrosis (Kharraz et al., 2013; Novak et al., 2014; Wang et al., 2014; Tonkin et al., 2015; Xiao et al., 2016a). Our previous studies and other studies have suggested that increased macrophage recruitment or macrophage depletion impairs skeletal muscle regeneration (Shen et al., 2008; Wang et al., 2014; Liu et al., 2016; Xiao et al., 2016a). Interestingly, we found that F4/80 and CD68, markers of macrophages, increased significantly 12 days (*p* < 0.01) and 24 days (*p* < 0.01) after contusion in the BMSCs treated group as compared with the vehicle treated group. Secondly, we found that BMSCs transplantation increased the expression of inflammatory cytokines (such as TNF-α, IL-1β, IFN-γ, IL-6 and TGF-β) in the later stage of skeletal muscle regeneration (Figure 7). Indeed, disease microenvironments have profound impacts on transplanted MSC sinmediating and modulating their therapeutic effects (Sui et al., 2017). Inflammatory cytokines (especially TGF-β) modulate MSC proliferation and myofibroblast differentiation. For example, in a study by Desai et al. (2014), adipose-derived mesenchymal stem cells (ADSCs) treated with TGF-β developed a myofibroblastic phenotype with increases in a-smooth muscle actin (a-SMA), a myofibroblast marker, and the ECM proteins type I collagen and fibronectin. di Bonzo et al. (2008) found that the differentiation of transplanted BMSCs, particularly under conditions of chronic injury, into pro-fibrogenic potential cells significantly contributed to liver fibrosis. In addition, Kim et al. (2014) found that normal human prostate-derived mesenchymal stem cells (MSCs) exposed to TGF-β1 can differentiate into myofibroblasts. Myofibroblasts are the primary extracellular matrix (ECM)-secreting cells during wound healing and fibrosis, and are largely responsible for scar tissue formation as the wound matures (Klingberg et al., 2013). Furthermore, in our previous study, we found that levels of pro-inflammatory cytokines (such as IL-1β, Il-6 and MCP-1) and MPO (a specific markers of neutrophils) were significantly increased at 6 h post-injury (Xiao et al., 2016b). Levels of the pro-inflammatory cytokines TGF-β, TNF-α and myostain were significantly increased 12 h post-injury (Xiao et al., 2016a). Taken together, these findings may suggest that pro-inflammatory microenvironments may induce BMSCs differentiation into myofibroblasts and impair skeletal muscle regeneration.

Moreover, we investigated muscle chemokines. The data showed that the expression pattern of chemokines, like inflammatory cytokines, increased significantly in the BMSCs treated mice in the later stage of muscle regeneration (Figure 8). CC chemokines are mainly involved in the recruitment of monocytes/macrophages, eosinophils, basophils, and lymphocytes, whereas CXC chemokines attract neutrophils to sites of injury (Boyd et al., 2006; Contreras-Shannon et al., 2007). In addition to their chemotactic effects on leukocytes, multiple chemokines have broader functions, such as influencing collagen production and proliferation of hematopoietic precursor cells (Warren et al., 2004). In previous studies, we showed that there was a high expression of chemokines in injured skeletal muscle with severe fibrosis (Xiao et al., 2016a). This result was similar to the outcome observed in the contused skeletal muscle of the BMSC treatment group. It has been suggested that BMSC transplantation impairs muscle regeneration and multiple chemokines may be involved.

In addition, we tested the expression of matrix metalloproteinases (MMPs), which are zinc-dependent endopeptidases that play an important role in the digestion of the ECM, inflammation and fibrosis in pathophysiological conditions (Kumar et al., 2010; Davis et al., 2013). Compared with the vehicle group, the transcription levels of MMPs were significantly upregulated in the BMSC-treated group after contusion (Figure 9). Interestingly, this phenomenon is similar to the process involved in other muscle disease models, such as dystrophic muscle. In dystrophic muscle of mdx mice, MMPs are significantly upregulated, whereas tissue inhibitors of MMPs are down regulated (Kumar et al., 2010). Deletion or inhibition of MMPs was found to dramatically improve muscle structure and function, as well as reduce muscle injury, inflammation and fiber necrosis in the muscle of mdx mice (Li et al., 2009; Hindi et al., 2013). In addition, BMSCs cocultured with C2C12 cells or their conditioned medium (MSC-CM) upregulated MMP-2 and MMP-9 expression (Sassoli et al., 2014). Moreover, we found that the increase in MMP (MMP-1, MMP-2, MMP-9, MMP-10 and MMP-14) levels for the BMSCs treated injury group is delayed compared to the MMP increase in the vehicle treated group. High MMPs expression impair muscle regeneration, and inhibition of MMPs using batimastat contributes muscle regeneration (Kumar et al., 2010; Ogura et al., 2014). These results suggest that MMPs may be involved in delayed skeletal muscle regeneration after BMSC transplantation. However, further studies need to explore the interaction between MMPs and MSCs and the mechanism involved in skeletal muscle regeneration.

**FIGURE 10:**
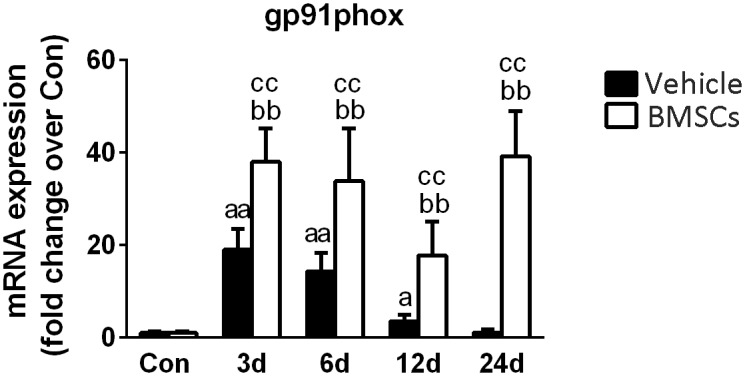
The effects of BMSCs treatment on the expression of gp91phox. Vehicle, muscle contusion and vehicle treated group; BMSCs, muscle contusion and BMSCs treated group. Data are means ± S.D., *n* = 8; ^a^Significant difference from control, *P* < 0.05; ^aa^*P* < 0.01. ^b^Significant difference from control, *P* < 0.05; ^bb^*P* < 0.01. ^c^Significant difference from the same time points of group vehicle, ^c^*P* < 0.05; ^cc^*P* < 0.01. BMSCs transplantation significantly enhanced the expression gp91phox in injured gastrocnemius muscle.

Next, we investigated gp91phox (formerly known as Nox2), which is a key membrane-bound subunit of NADPH oxidase and is used as a marker of NADPH oxidase (Ghaly and Marsh, 2010; Xiao et al., 2012; Liu et al., 2018). It has been acknowledged that NADPH oxidase is a primary source of ROS generation, and the consequential of oxidative stress in various tissues (Chan et al., 2009). Recently, Rabani et al. (2018) found that when BMSCs cocultured with macrophages, BMSC-induced ROS production in macrophages is dependent on the activation of gp91phox. In this study, the expression of gp91phox increased significantly after muscle contusion and returned to normal at 24 days post-injury. However, BMSC transplantation significantly upregulated the expression of gp91phox after injury compared with the gp91phox expression in the vehicle group (*p* < 0.01) (Figure 10). Similar results were seen in other disease models. Compared to wild mice, gp91phox levels were significantly increased in the tibialis anterior muscles of mdx mice (Whitehead et al., 2010). Likewise, pharmacologically induced liver fibrosis was attenuated in gp91phox-deficient mice (Novitskiy et al., 2006). Moreover, administration of apocynin (the NADPH oxidase inhibitor) suppressed the development of renal fibrosis hypertensive rats (Zhao et al., 2008). These results indicate that BMSC transplantation impairs muscle regeneration and that NADPH oxidase may play an important role in this process.

These results may be different from the results of other studies, that found that mesenchymal stem cells [BMSCs (von Roth et al., 2012a; Winkler et al., 2012), adipose MSCs, embryonic stem cells (Ninagawa et al., 2013), umbilical cord MSCs (Grabowska et al., 2013) and skeletal muscle MSCs (Meligy et al., 2012)] contribute to muscle regeneration and improve muscle force after injury. However, in other disease models [such as lung injury (Yao et al., 2018) and various cancers (Norozi et al., 2016; Lee and Hong, 2017), including leukemia (Low et al., 2015), and hepatocarcinoma (Zong et al., 2018)], BMSC transplantation aggravated the disease condition that was regulated by disease microenvironments. In this study, we first found BMSC transplantation impaired muscle regeneration after muscle contusion.

## Conclusion

Our results suggest that BMSC transplantation induced impaired skeletal muscle regeneration and that macrophages, inflammatory cytokines, chemokines, matrix metalloproteinases and oxidative stress-related enzymes may be involved in the process. These findings shed new light on the role of BMSC in regenerative medicine and indicate that caution is needed in the application of BMSCs for muscle injury.

## Consent for Publication

All authors consent to publication.

## Availability of Data and Materials

All data generated and/or analyzed during this study are included in this published article.

## Author Contributions

WX and PC designed this study and helped to draft the manuscript. XL carried out data analysis and drafted the manuscript. LZ and YZ performed the isolation and cultured BMSCs. XL, LZ, and YZ performed the histological staining and carried out the real time PCR. All authors have read and approved the final version of the manuscript, and agreed with the order of presentation of the authors.

## Conflict of Interest Statement

The authors declare that the research was conducted in the absence of any commercial or financial relationships that could be construed as a potential conflict of interest.

## Abbreviations

BMSCs: Bone marrow mesenchymal stem cells
CCL2: Chemokine (C-C motif) ligand 2
CCL5: Chemokine (C-C motif) ligand 5
CCR2: C-C chemokine receptor type 2
CXCR4: C-X-C chemokine receptor type 4
Fn14: fibroblast growth factor-inducible protein 14
GMs: gastrocnemius muscles
H&E: Hematoxylin and Eosin
IFN-γ: Interferon gamma
IL-1β: Interleukin 1 beta
IL-6: Interleukin 6
MMP-1: Metalloproteinase-1
MMP-2: Metalloproteinase-2
MMP-9: Metalloproteinase-9
MMP-10: Metalloproteinase-10
MMP-14: Metalloproteinase-14
PBS: phosphate buffer solution
RT-PCR: Real-Time Polymerase Chain Reaction
TGF-β: Transforming growth factor beta
TWEAK: TNF-related weak inducer of apoptosis.

## References

[B1] Aldahmash A. Zaher W. Al-Nbaheen M. Kassem M. (2012). Human stromal (mesenchymal) stem cells: basic biology and current clinical use for tissue regeneration. *Ann. Saudi Med.* 32 68–77. 10.5144/0256-4947.2012.68 22156642 PMC6087654

[B2] Bashir J. Sherman A. Lee H. Kaplan L. Hare J. M. (2014). Mesenchymal stem cell therapies in the treatment of musculoskeletal diseases. *PMR* 6 61–69. 10.1016/j.pmrj.2013.05.007 24439148

[B3] Boyd J. H. Divangahi M. Yahiaoui L. Gvozdic D. Qureshi S. Petrof B. J. (2006). Toll-like receptors differentially regulate CC and CXC chemokines in skeletal muscle via NF-kappaB and calcineurin. *Infect. Immun.* 74 6829–6838. 10.1128/IAI.00286-06 16982839 PMC1698076

[B4] Carvalho A. B. Quintanilha L. F. Dias J. V. Paredes B. D. Mannheimer E. G. Carvalho F. G. (2008). Bone marrow multipotent mesenchymal stromal cells do not reduce fibrosis or improve function in a rat model of severe chronic liver injury. *Stem Cells* 26 1307–1314. 10.1634/stemcells.2007-0941 18308943

[B5] Chan E. C. Jiang F. Peshavariya H. M. Dusting G. J. (2009). Regulation of cell proliferation by NADPH oxidase-mediated signaling: potential roles in tissue repair, regenerative medicine and tissue engineering. *Pharmacol. Ther.* 122 97–108. 10.1016/j.pharmthera.2009.02.005 19285105

[B6] Contreras-Shannon V. Ochoa O. Reyes-Reyna S. M. Sun D. Michalek J. E. Kuziel W. A. (2007). Fat accumulation with altered inflammation and regeneration in skeletal muscle of CCR2-/- mice following ischemic injury. *Am. J. Physiol. Cell. Physiol.* 292 C953–C967. 10.1152/ajpcell.00154.2006 17020936

[B7] da Silva R. P. Gordon S. (1999). Phagocytosis stimulates alternative glycosylation of macrosialin (mouse CD68), a macrophage-specific endosomal protein. *Biochem. J.* 338( Pt 3), 687–694. 10051440 PMC1220104

[B8] Davis M. E. Gumucio J. P. Sugg K. B. Bedi A. Mendias C. L. (2013). MMP inhibition as a potential method to augment the healing of skeletal muscle and tendon extracellular matrix. *J. Appl. Physiol.* 115 884–891. 10.1152/japplphysiol.00137.2013 23640595 PMC3764625

[B9] de la Garza-Rodea A. S. van der Velde I. Boersma H. Goncalves M. A. van Bekkum D. W. de Vries A. A. (2011). Long-term contribution of human bone marrow mesenchymal stromal cells to skeletal muscle regeneration in mice. *Cell Transplant.* 20 217–231. 10.3727/096368910X522117 20719081

[B10] Desai V. D. Hsia H. C. Schwarzbauer J. E. (2014). Reversible modulation of myofibroblast differentiation in adipose-derived mesenchymal stem cells. *PLoS One* 9:e86865. 10.1371/journal.pone.0086865 24466271 PMC3900664

[B11] Dezawa M. Ishikawa H. Itokazu Y. Yoshihara T. Hoshino M. Takeda S. (2005). Bone marrow stromal cells generate muscle cells and repair muscle degeneration. *Science* 309 314–317. 10.1126/science.1110364 16002622

[B12] di Bonzo L. V. Ferrero I. Cravanzola C. Mareschi K. Rustichell D. Novo E. (2008). Human mesenchymal stem cells as a two-edged sword in hepatic regenerative medicine: engraftment and hepatocyte differentiation versus profibrogenic potential. *Gut* 57 223–231. 10.1136/gut.2006.111617 17639088

[B13] Dominici M. Le Blanc K. Mueller I. Slaper-Cortenbach I. Marini F. Krause D. (2006). Minimal criteria for defining multipotent mesenchymal stromal cells. The International Society for Cellular Therapy position statement. *Cytotherapy* 8 315–317. 10.1080/14653240600855905 16923606

[B14] Farjah G. H. Fazli F. Karimipour M. Pourheidar B. Heshmatiyan B. Pourheidar M. (2018). The effect of bone marrow mesenchymal stem cells on recovery of skeletal muscle after neurotization surgery in rat. *Iran. J. Basic Med. Sci.* 21 236–243. 10.22038/ijbms.2018.22327.5699 29511489 PMC5817166

[B15] Galli D. Vitale M. Vaccarezza M. (2014). Bone marrow-derived mesenchymal cell differentiation toward myogenic lineages: facts and perspectives. *Biomed. Res. Int.* 2014:762695. 10.1155/2014/762695 25054145 PMC4099119

[B16] Geng J. Peng F. Xiong F. Shang Y. Zhao C. Li W. (2009). Inhibition of myostatin promotes myogenic differentiation of rat bone marrow-derived mesenchymal stromal cells. *Cytotherapy* 11 849–863. 10.3109/14653240903131632 19903098

[B17] Ghaly A. Marsh D. R. (2010). Aging-associated oxidative stress modulates the acute inflammatory response in skeletal muscle after contusion injury. *Exp. Gerontol.* 45 381–388. 10.1016/j.exger.2010.03.004 20211238

[B18] Grabowska I. Streminska W. Janczyk-Ilach K. Machaj E. K. Pojda Z. Hoser G. (2013). Myogenic potential of mesenchymal stem cells - the case of adhesive fraction of human umbilical cord blood cells. *Curr. Stem Cell Res. Ther.* 8 82–90. 10.2174/1574888X11308010010 23270632

[B19] Hindi S. M. Shin J. Ogura Y. Li H. Kumar A. (2013). Matrix metalloproteinase-9 inhibition improves proliferation and engraftment of myogenic cells in dystrophic muscle of mdx mice. *PLoS One* 8:e72121. 10.1371/journal.pone.0072121 23977226 PMC3744489

[B20] Huebner K. D. Jassal D. S. Halevy O. Pines M. Anderson J. E. (2008). Functional resolution of fibrosis in mdx mouse dystrophic heart and skeletal muscle by halofuginone. *Am. J. Physiol. Heart Circ. Physiol.* 294 H1550–H1561. 10.1152/ajpheart.01253.2007 18263710

[B21] Kharraz Y. Guerra J. Mann C. J. Serrano A. L. Munoz-Canoves P. (2013). Macrophage plasticity and the role of inflammation in skeletal muscle repair. *Mediators Inflamm.* 2013:491497. 10.1155/2013/491497 23509419 PMC3572642

[B22] Kim W. Barron D. A. San Martin R. Chan K. S. Tran L. L. Yang F. (2014). RUNX1 is essential for mesenchymal stem cell proliferation and myofibroblast differentiation. *Proc. Natl. Acad. Sci. U.S.A.* 111 16389–16394. 10.1073/pnas.1407097111 25313057 PMC4246299

[B23] Klingberg F. Hinz B. White E. S. (2013). The myofibroblast matrix: implications for tissue repair and fibrosis. *J. Pathol.* 229 298–309. 10.1002/path.4104 22996908 PMC4005341

[B24] Kumar A. Bhatnagar S. Kumar A. (2010). Matrix metalloproteinase inhibitor batimastat alleviates pathology and improves skeletal muscle function in dystrophin-deficient mdx mice. *Am. J. Pathol.* 177 248–260. 10.2353/ajpath.2010.091176 20472898 PMC2893668

[B25] Lee H. Y. Hong I. S. (2017). Double-edged sword of mesenchymal stem cells: cancer-promoting versus therapeutic potential. *Cancer Sci.* 108 1939–1946. 10.1111/cas.13334 28756624 PMC5623746

[B26] Leroux L. Descamps B. Tojais N. F. Seguy B. Oses P. Moreau C. (2010). Hypoxia preconditioned mesenchymal stem cells improve vascular and skeletal muscle fiber regeneration after ischemia through a Wnt4-dependent pathway. *Mol. Ther.* 18 1545–1552. 10.1038/mt.2010.108 20551912 PMC2927059

[B27] Li H. Mittal A. Makonchuk D. Y. Bhatnagar S. Kumar A. (2009). Matrix metalloproteinase-9 inhibition ameliorates pathogenesis and improves skeletal muscle regeneration in muscular dystrophy. *Hum. Mol. Genet.* 18 2584–2598. 10.1093/hmg/ddp191 19401296 PMC2701330

[B28] Lieber R. L. Ward S. R. (2013). Cellular mechanisms of tissue fibrosis. 4. Structural and functional consequences of skeletal muscle fibrosis. *Am. J. Physiol. Cell. Physiol.* 305 C241–C252. 10.1152/ajpcell.00173.2013 23761627 PMC3742845

[B29] Liu X. Liu Y. Zhao L. Zeng Z. Xiao W. Chen P. (2016). Macrophage depletion impairs skeletal muscle regeneration: the roles of regulatory factors for muscle regeneration. *Cell Biol. Int.* 41 228–238. 10.1002/cbin.10705 27888539

[B30] Liu X. Zeng Z. Zhao L. Xiao W. Chen P. (2018). Changes in inflammatory and oxidative stress factors and the protein synthesis pathway in injured skeletal muscle after contusion. *Exp. Ther. Med.* 15 2196–2202. 10.3892/etm.2017.5625 29434825 PMC5776558

[B31] Low J. H. Ramdas P. Radhakrishnan A. K. (2015). Modulatory effects of mesenchymal stem cells on leucocytes and leukemic cells: a double-edged sword? *Blood Cells Mol. Dis.* 55 351–357. 10.1016/j.bcmd.2015.07.017 26460259

[B32] Meligy F. Y. Shigemura K. Behnsawy H. M. Fujisawa M. Kawabata M. Shirakawa T. (2012). The efficiency of in vitro isolation and myogenic differentiation of MSCs derived from adipose connective tissue, bone marrow, and skeletal muscle tissue. *In Vitro Cell. Dev. Biol. Anim.* 48 203–215. 10.1007/s11626-012-9488-x 22396125

[B33] Merritt E. K. Cannon M. V. Hammers D. W. Le L. N. Gokhale R. Sarathy A. (2010). Repair of traumatic skeletal muscle injury with bone-marrow-derived mesenchymal stem cells seeded on extracellular matrix. *Tissue Eng. Part A* 16 2871–2881. 10.1089/ten.TEA.2009.0826 20412030

[B34] Natsu K. Ochi M. Mochizuki Y. Hachisuka H. Yanada S. Yasunaga Y. (2004). Allogeneic bone marrow-derived mesenchymal stromal cells promote the regeneration of injured skeletal muscle without differentiation into myofibers. *Tissue Eng.* 10 1093–1112. 10.1089/ten.2004.10.1093 15363167

[B35] Ninagawa N. T. Isobe E. Hirayama Y. Murakami R. Komatsu K. Nagai M. (2013). Transplantated mesenchymal stem cells derived from embryonic stem cells promote muscle regeneration and accelerate functional recovery of injured skeletal muscle. *Biores. Open Access* 2 295–306. 10.1089/biores.2013.0012 23914336 PMC3731682

[B36] Norozi F. Ahmadzadeh A. Shahrabi S. Vosoughi T. Saki N. (2016). Mesenchymal stem cells as a double-edged sword in suppression or progression of solid tumor cells. *Tumour Biol.* 37 11679–11689. 10.1007/s13277-016-5187-7 27440203

[B37] Novak M. L. Weinheimer-Haus E. M. Koh T. J. (2014). Macrophage activation and skeletal muscle healing following traumatic injury. *J. Pathol.* 232 344–355. 10.1002/path.4301 24255005 PMC4019602

[B38] Novitskiy G. Potter J. J. Wang L. Mezey E. (2006). Influences of reactive oxygen species and nitric oxide on hepatic fibrogenesis. *Liver Int.* 26 1248–1257. 10.1111/j.1478-3231.2006.01364.x 17105591

[B39] Ogura Y. Tajrishi M. M. Sato S. Hindi S. M. Kumar A. (2014). Therapeutic potential of matrix metalloproteinases in Duchenne muscular dystrophy. *Front. Cell Dev. Biol.* 2:11 10.3389/fcell.2014.00011 PMC4207008 25364719

[B40] Rabani R. Volchuk A. Jerkic M. Ormesher L. Garces-Ramirez L. Canton J. (2018). Mesenchymal stem cells enhance NOX2 dependent ROS production and bacterial killing in macrophages during sepsis. *Eur. Respir. J.* 51:1702021. 10.1183/13993003.02021-2017 29519920

[B41] Sassoli C. Nosi D. Tani A. Chellini F. Mazzanti B. Quercioli F. (2014). Defining the role of mesenchymal stromal cells on the regulation of matrix metalloproteinases in skeletal muscle cells. *Exp. Cell Res.* 323 297–313. 10.1016/j.yexcr.2014.03.003 24631289

[B42] Sassoli C. Pini A. Chellini F. Mazzanti B. Nistri S. Nosi D. (2012). Bone marrow mesenchymal stromal cells stimulate skeletal myoblast proliferation through the paracrine release of VEGF. *PLoS One* 7:e37512. 10.1371/journal.pone.0037512 22815682 PMC3398011

[B43] Shen W. Li Y. Zhu J. Schwendener R. Huard J. (2008). Interaction between macrophages, TGF-beta1, and the COX-2 pathway during the inflammatory phase of skeletal muscle healing after injury. *J. Cell. Physiol.* 214 405–412. 10.1002/jcp.21212 17657727

[B44] Starkey P. M. Turley L. Gordon S. (1987). The mouse macrophage-specific glycoprotein defined by monoclonal antibody F4/80: characterization, biosynthesis and demonstration of a rat analogue. *Immunology* 60 117–122. 3817865 PMC1453359

[B45] Su J. Chen X. Huang Y. Li W. Li J. Cao K. (2014). Phylogenetic distinction of iNOS and IDO function in mesenchymal stem cell-mediated immunosuppression in mammalian species. *Cell Death Differ.* 21 388–396. 10.1038/cdd.2013.149 24162664 PMC3921585

[B46] Sui B. D. Hu C. H. Liu A. Q. Zheng C. X. Xuan K. Jin Y. (2017). Stem cell-based bone regeneration in diseased microenvironments: Challenges and solutions. *Biomaterials* 10.1016/j.biomaterials.2017.10.046 [Epub ahead of print]. 29122279

[B47] Tonkin J. Temmerman L. Sampson R. D. Gallego-Colon E. Barberi L. Bilbao D. (2015). Monocyte/macrophage-derived IGF-1 orchestrates murine skeletal muscle regeneration and modulates autocrine polarization. *Mol. Ther.* 23 1189–1200. 10.1038/mt.2015.66 25896247 PMC4817788

[B48] von Roth P. Duda G. N. Radojewski P. Preininger B. Perka C. Winkler T. (2012a). Mesenchymal stem cell therapy following muscle trauma leads to improved muscular regeneration in both male and female rats. *Gend. Med.* 9 129–136. 10.1016/j.genm.2012.01.007 22361839

[B49] von Roth P. Duda G. N. Radojewski P. Preininger B. Strohschein K. Rohner E. (2012b). Intra-arterial MSC transplantation restores functional capacity after skeletal muscle trauma. *Open Orthop. J.* 6 352–356. 10.2174/1874325001206010352 22927895 PMC3426785

[B50] von Roth P. Winkler T. Rechenbach K. Radojewski P. Perka C. Duda G. N. (2013). Improvement of contraction force in injured skeletal muscle after autologous mesenchymal stroma cell transplantation is accompanied by slow to fast fiber type shift. *Transfus. Med. Hemother.* 40 425–430. 10.1159/000354127 24474893 PMC3901591

[B51] Wang H. Melton D. W. Porter L. Sarwar Z. U. McManus L. M. Shireman P. K. (2014). Altered macrophage phenotype transition impairs skeletal muscle regeneration. *Am. J. Pathol.* 184 1167–1184. 10.1016/j.ajpath.2013.12.020 24525152 PMC3969996

[B52] Warren G. L. O’Farrell L. Summan M. Hulderman T. Mishra D. Luster M. I. (2004). Role of CC chemokines in skeletal muscle functional restoration after injury. *Am. J. Physiol. Cell. Physiol.* 286 C1031–C1036. 10.1152/ajpcell.00467.2003 15075201

[B53] Whitehead N. P. Yeung E. W. Froehner S. C. Allen D. G. (2010). Skeletal muscle NADPH oxidase is increased and triggers stretch-induced damage in the mdx mouse. *PLoS One* 5:e15354. 10.1371/journal.pone.0015354 21187957 PMC3004864

[B54] Winkler T. von Roth P. Matziolis G. Mehta M. Perka C. Duda G. N. (2009). Dose-response relationship of mesenchymal stem cell transplantation and functional regeneration after severe skeletal muscle injury in rats. *Tissue Eng. Part A* 15 487–492. 10.1089/ten.tea.2007.0426 18673090

[B55] Winkler T. von Roth P. Radojewski P. Urbanski A. Hahn S. Preininger B. (2012). Immediate and delayed transplantation of mesenchymal stem cells improve muscle force after skeletal muscle injury in rats. *J. Tissue Eng. Regen. Med.* 6(Suppl. 3), s60–s67. 10.1002/term.1542 22761111

[B56] Winkler T. von Roth P. Schumann M. R. Sieland K. Stoltenburg-Didinger G. Taupitz M. (2008). In vivovisualization of locally transplanted mesenchymal stem cells in the severely injured muscle in rats. *Tissue Eng. Part A* 14 1149–1160. 10.1089/ten.tea.2007.0179 18433314

[B57] Xiao W. Chen P. Dong J. (2012). Effects of overtraining on skeletal muscle growth and gene expression. *Int. J. Sports Med.* 33 846–853. 10.1055/s-0032-1311585 22592543

[B58] Xiao W. Chen P. Wang R. Dong J. (2013). Overload training inhibits phagocytosis and ROS generation of peritoneal macrophages: role of IGF-1 and MGF. *Eur. J. Appl. Physiol.* 113 117–125. 10.1007/s00421-012-2418-5 22592456

[B59] Xiao W. Liu Y. Chen P. (2016a). Macrophage depletion impairs skeletal muscle regeneration: the roles of pro-fibrotic factors, inflammation, and oxidative stress. *Inflammation* 39 2016–2028. 10.1007/s10753-016-0438-8 27605219

[B60] Xiao W. Liu Y. Luo B. Zhao L. Liu X. Zeng Z. (2016b). Time-dependent gene expression analysis after mouse skeletal muscle contusion. *J. Sport Health Sci.* 5 101–108. 10.1016/j.jshs.2016.01.017 30356928 PMC6191981

[B61] Yao Y. Zheng Z. Song Q. (2018). Mesenchymal stem cells: A double-edged sword in radiation-induced lung injury. *Thorac. Cancer* 9 208–217. 10.1111/1759-7714.12573 29235254 PMC5792737

[B62] Zhao W. Chen S. S. Chen Y. Ahokas R. A. Sun Y. (2008). Kidney fibrosis in hypertensive rats: role of oxidative stress. *Am. J. Nephrol.* 28 548–554. 10.1159/000115289 18239381 PMC2821447

[B63] Zong C. Zhang H. Yang X. Gao L. Hou J. Ye F. (2018). The distinct roles of mesenchymal stem cells in the initial and progressive stage of hepatocarcinoma. *Cell Death Dis.* 9:345. 10.1038/s41419-018-0366-7 29497038 PMC5832809

