# Corrigendum: BMSC Transplantation Aggravates Inflammation, Oxidative Stress, and Fibrosis and Impairs Skeletal Muscle Regeneration

**DOI:** 10.3389/fphys.2019.01154

**Published:** 2019-09-11

**Authors:** Xiaoguang Liu, Lifang Zheng, Yongzhan Zhou, Yingjie Chen, Peijie Chen, Weihua Xiao

**Affiliations:** School of Kinesiology, Shanghai University of Sport, Shanghai, China

**Keywords:** bone marrow mesenchymal stem cells, skeletal muscle, regeneration, inflammation, oxidative stress, fibrosis

An author name was incorrectly spelled as “Lifang Zhen.” The correct spelling is “Lifang Zheng.”

Additionally, in the original article, there was an error. The sentence indicating that two authors contributed equally to the work was erroneously omitted.

A correction has been made to the cover page of the article:

“These authors have contributed equally to this work”

The authors apologize for this error and state that this does not change the scientific conclusions of the article in any way. The original article has been updated.

